# Decreasing surface albedo signifies a growing importance of clouds for Greenland Ice Sheet meltwater production

**DOI:** 10.1038/s41467-022-31434-w

**Published:** 2022-07-21

**Authors:** J. C. Ryan, L. C. Smith, S. W. Cooley, B. Pearson, N. Wever, E. Keenan, J. T. M. Lenaerts

**Affiliations:** 1 Department of Geography, University of Oregon, Eugene, OR USA; 2 Institute at Brown for Environment and Society, Brown University, Providence, RI USA; 3 Department of Earth, Environmental and Planetary Sciences, Brown University, Providence, RI USA; 4 College of Earth, Ocean, and Atmospheric Sciences, Oregon State University, Corvallis, OR USA; 5 Department of Atmospheric and Oceanic Sciences, University of Colorado, Boulder, CO USA

**Keywords:** Cryospheric science, Projection and prediction

## Abstract

Clouds regulate the Greenland Ice Sheet’s surface energy balance through the competing effects of shortwave radiation shading and longwave radiation trapping. However, the relative importance of these effects within Greenland’s narrow ablation zone, where nearly all meltwater runoff is produced, remains poorly quantified. Here we use machine learning to merge MODIS, CloudSat, and CALIPSO satellite observations to produce a high-resolution cloud radiative effect product. For the period 2003–2020, we find that a 1% change in cloudiness has little effect (±0.16 W m^−2^) on summer net radiative fluxes in the ablation zone because the warming and cooling effects of clouds compensate. However, by 2100 (SSP5-8.5 scenario), radiative fluxes in the ablation zone will become more than twice as sensitive (±0.39 W m^−2^) to changes in cloudiness due to reduced surface albedo. Accurate representation of clouds will therefore become increasingly important for forecasting the Greenland Ice Sheet’s contribution to global sea-level rise.

## Abstract (web summary)

Here the authors use remote sensing observations and machine learning to show that clouds will become increasingly important for determining the Greenland Ice Sheet’s contribution to global sea levels due to decreasing albedo in the ablation zone.

## Introduction

The Greenland Ice Sheet has been losing mass since the 1990s and is currently the single largest cryospheric contributor to observed global sea-level rise^1^. This recent mass loss is primarily due to enhanced surface meltwater runoff, most of which derives from the narrow ablation zone where bare, dark glacial ice is exposed each summer^2,3^. There is a growing realization that clouds play an important role in determining the amount of meltwater runoff generated in the ablation zone^4–7^. Clouds are known to regulate surface energy balance through the competing effects of shortwave radiation shading (i.e. cooling) and longwave radiation trapping (i.e. warming)^4,7–9^. However, the precise radiative effects of clouds in the ablation zone remains uncertain due to a reliance on modeled radiative fluxes which are known to contain biases^8,10,11^. Therefore, it remains largely unknown how changes in cloudiness, such as those which occur due to atmospheric blocking events^12^, alter the rate of meltwater production from the ablation zone both currently and in the future.

While in situ observations of cloud radiative effects (CRE) have been acquired at research stations, they are sparsely distributed and mainly constrained to the accumulation zone (e.g.^13–16^. Satellite remote sensing therefore provides the only practicable technique for observing CRE across the whole ice sheet. A few studies have used CloudSat and Cloud-Aerosol Lidar and Infrared Pathfinder Satellite Observation (CALIPSO) to characterize cloud properties and their radiative effects over Greenland and the Arctic^7,8,17,18^. However, the active remote sensing instruments employed on these satellites have sparse spatiotemporal sampling, requiring aggregation into very coarse grid cells (e.g. 2 × 2° or 2 × 1°) to ensure a sufficient number of samples within each grid cell. Such products are therefore too coarse to resolve Greenland’s narrow ablation zone^7,8,11^, preventing characterization of CRE for the very part of the ice sheet that generates runoff. Optical remote sensing instruments such as the MODerate Imaging Spectroradiometer (MODIS) onboard Terra and Aqua offer much finer spatial and temporal resolution^19^. Yet visible-infrared wavelengths cannot provide a complete vertical profile of cloud structure, meaning that MODIS-derived cloud properties and associated radiative effects are inherently uncertain. The production of high-quality CRE observations at high spatial resolution therefore requires intelligent integration of cloud profiling with optical imagery, to combine the strengths of these different sensor technologies while overcoming their individual limitations.

Such a task is well-suited to supervised machine learning techniques that can identify complex, non-linear relationships in data to make predictions without explicit consideration of the intermediate physical processes. Machine learning has been particularly successful for integrating sparse, high-quality field measurements with more extensive remotely sensed observations for the prediction of phenomena across large spatial scales^20^. This approach has been widely used in the geosciences for predicting land cover^21^, evapotranspiration^22^, and CO_2_ emissions^23^. However, machine learning is relatively undeveloped in glaciology and, to our knowledge, has not been used to combine remote sensing data for investigations of Greenland Ice Sheet surface energy balance.

Here we use Random Forest Regression to fuse the respective strengths of CloudSat/CALIPSO (high accuracy vertical profiles of cloud properties) and MODIS (high spatial and temporal resolution visible-infrared imagery), enabling accurate, daily quantification of CRE over the Greenland Ice Sheet during summer for the 2003–2020 period (see Methods). We then exploit the unprecedented spatial resolution of our product (1 × 1 km grid cell size) to resolve fine spatial gradients and interannual variations of CRE within the narrow ice sheet ablation zone, where nearly all meltwater runoff is produced. We also use our product to investigate the impacts of anticyclonic circulation regimes (i.e. blocking events) on clouds and radiative fluxes in the ablation zone. Finally, we combine our product with projections from the CMIP6 experiment to assess the radiative effects of clouds and atmospheric blocking events on Greenland Ice Sheet surface energy balance in a future, warmer climate.

## Results

### Cloud radiative effects observed at high-resolution

**Fig. 1:**
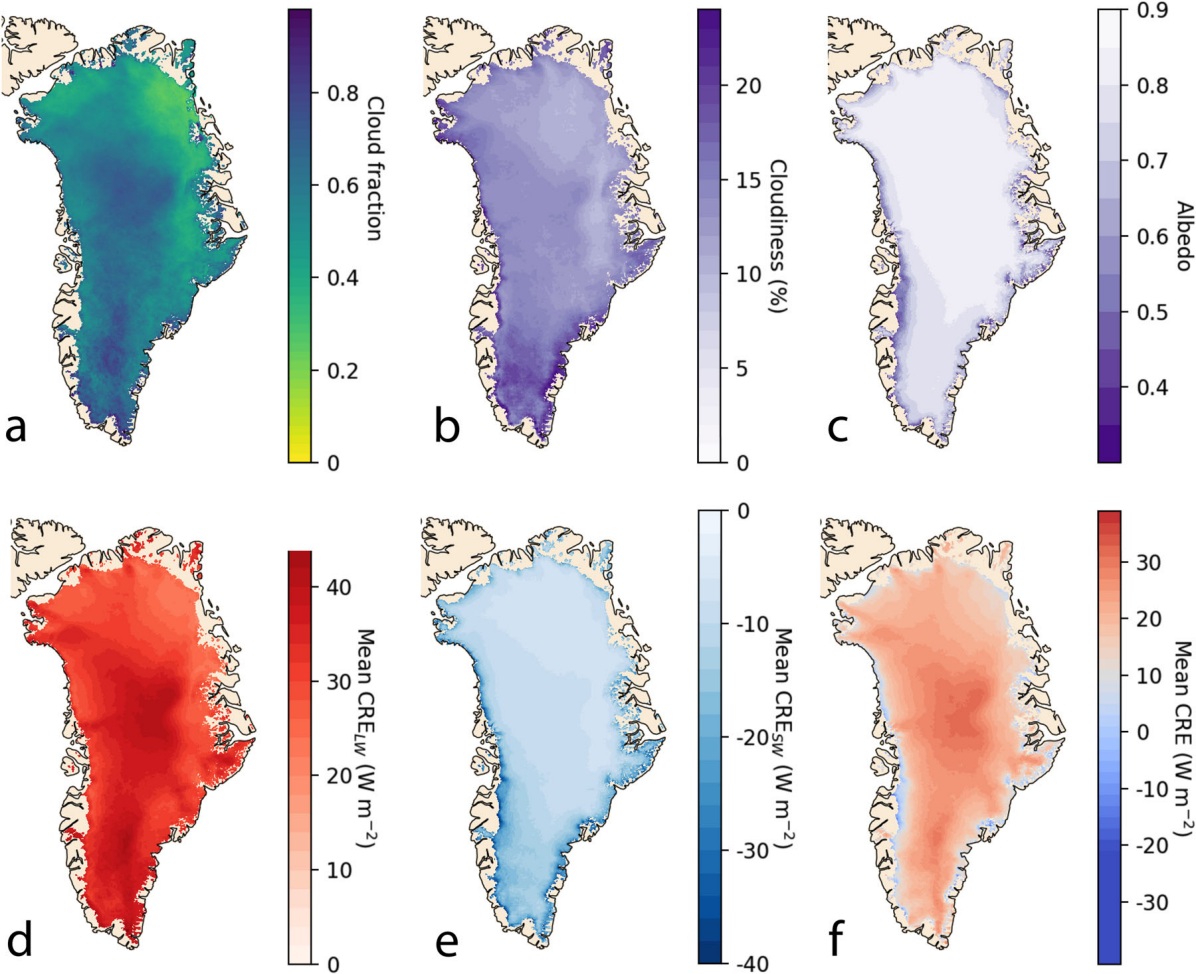
High-resolution (1 km × 1 km) predictions of summer cloudiness, albedo and cloud radiative effects (CRE) over the Greenland Ice Sheet derived from fusing different satellite datasets with machine learning. **a** cloud fraction, defined as the frequency of cloud cover (**b**) cloudiness, defined as the percentage reduction of shortwave radiative fluxes at the surface due to clouds; (**c**) surface albedo; (**d**) cloud radiative effect on shortwave radiation received at the ice surface; (**e**) cloud radiative effect on longwave radiation received at the surface; (**f**) cloud radiative effect on net all-wave radiation at the surface. This product was derived by fusing CloudSat/CALIPSO radiative flux and MODIS cloud and surface albedo products.

We find that, averaged across the ice sheet, mean summer cloud cover is 54% for the 2003–2020 period, varying from 32% (5^th^ percentile) in the Northeast Greenland to 71% (95^th^ percentile) in South Greenland (Fig. 1a). Relative to clear skies, we find that clouds increase summer downward longwave radiation received at the surface by 16.4 ± 2.0% and decrease summer downward shortwave radiation received at the surface by 13.6 ± 2.6% (Fig. 1d, e). When these two competing effects are combined, we find that clouds exert a net warming effect on the Greenland Ice Sheet of +21.8 ± 5.9 W m^−2^ during the summer. These averaged values, however, mask strong spatial variations in CRE, which have a much larger warming effect in the accumulation zone (+23.5 ± 6.3 W m^−2^) than the ablation zone (+11.4 ± 3.1 W m^−2^), a finding that corroborates previous studies^6,16^ (Fig. 1f, S1). This can be attributed to lower albedo in the ablation zone (Fig. 1c), which amplifies the shortwave shading effect of clouds (Fig. 1d). Within the ablation zone, our high-resolution CRE product reveals that clouds have both a cooling and warming effect on average during our 18-year study period (Fig. 1f). CRE is negative near the lower elevation margins of the ablation zone (16% of the ablation zone area) but positive in the upper elevations of the ablation zone (84% of the ablation zone area; S1). These steep spatial gradients likely explain why previous studies have arrived at different conclusions about the role of clouds on ice sheet melt^5,7^.

Although we find no statistically significant trends (either regionally or at the ice sheet scale) during our 2003–2020 study period, we find substantial interannual variability (std. dev. = ±1.6%) in summer “cloudiness” (Fig. S2a, b; defined as the percentage reduction of shortwave radiative fluxes at the surface due to clouds, see Methods). Cloudiness ranges from as high as 16.2% in the summer of 2018 to as low as 10.8% in the summer of 2015. Temporal variability in summer cloudiness is somewhat higher in the ablation zone (std. dev. = ±2.3%) than in the accumulation zone (std. dev. = ±1.5%). We find no significant correlation between summer cloudiness and mean summer air temperatures in the ablation zone (Fig. S3a and S4), indicating that cloudiness likely will not change substantially in a warmer climate. We also observe substantial interannual variability in summer albedo which ranges by 4.0% with much more variation in the ablation zone (std. dev. = ±3.4%) than the accumulation zone (std. dev. = ±0.7%) (Fig. S2c, d). Variations in albedo can be attributed to processes such as snowline fluctuations, ice algae growth, meltwater ponding, surface roughening, and cryoconite hole expansion, all of which are captured by the high-resolution remotely sensed albedo products used in this study (see Methods^2,24^. Unlike cloudiness, we find that mean summer albedo is strongly linearly correlated with mean summer air temperatures over our 2003–2020 study period (*R*^2^ = 0.76), indicating that the ablation zone will darken in a warmer climate (Fig. S3c).

**Fig. 2:**
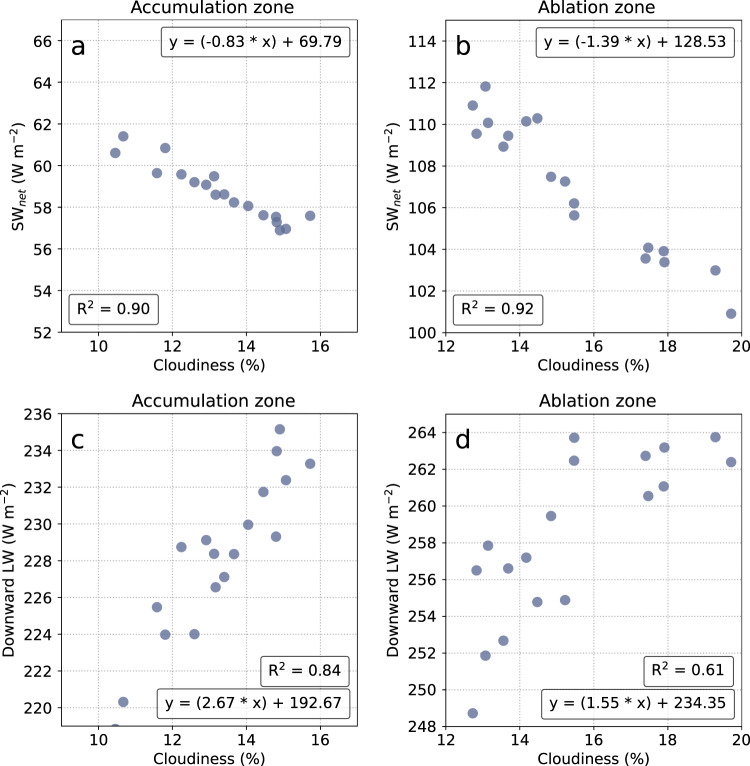
Correlative relationships between summer cloudiness and radiative fluxes at the ice sheet surface. **a** Net shortwave response to cloudiness in the accumulation zone, (**b**) Net shortwave response to cloudiness in the ablation zone, (**c**) Downward longwave response to cloudiness in the accumulation zone, (**d**) Downward longwave response to cloudiness in the ablation zone. Note that x and y axes are scaled to the same range. The slopes of these relationships signify the sensitivity of radiative fluxes to changes in summer cloudiness.

As expected, we find a strong positive correlation between cloudiness and downward longwave radiative fluxes and a strong negative correlation between cloudiness and downward shortwave radiative fluxes in our high-resolution CRE product (Fig. 2). In the high-albedo accumulation zone, the net effect is that cloud longwave trapping overwhelms cloud shortwave shading. For every 1% change in summer cloudiness, downward longwave radiation changes by 2.67 W m^−2^ while net shortwave radiation changes by only 0.83 W m^−2^ (Fig. 2a, c). The ~1.8 W m^−2^ difference between the two competing effects signifies that variations in cloudiness exert an important control on the surface energy balance of the accumulation zone. However, since only a small amount of meltwater runoff is produced from the accumulation zone^3^, this finding has little or no effect on Greenland’s contribution to global sea-level.

Interestingly, we also find that clouds have relatively little impact on meltwater production in the ablation zone, but for different reasons than the accumulation zone. During our study period, the shortwave shading response to changing cloudiness is approximately equal to the corresponding response of longwave trapping (±1.39 W m^−2^ and ±1.55 W m^−2^, respectively) (Fig. 2b, d). The compensating effects of clouds (when averaged across the entire ablation zone) signify that variations in summer cloudiness currently only have a small effect (±0.16 W m^−2^) on surface energy balance and associated meltwater production from the Greenland Ice Sheet. This finding is supported by the fact that intense melt years (e.g. 2010, 2012, and 2019) were not significantly cloudier/clearer than low melt years (e.g. 2013, 2015) (Fig. S3a).

### Cloud radiative effects during atmospheric blocking events

Since MODIS has daily temporal resolution, our high-resolution product enables investigation of the radiative effects of short-lived atmospheric circulation features occurring for days-to-weeks over Greenland. One of the most important of these for ice sheet surface melt is anticyclonic blocking events, which enhance ice sheet melt through cloud suppression and warm air advection^25^. Blocking events have been attributed to negative phases of the North Atlantic Oscillation (NAO) and have significantly increased in frequency over the past twenty years^26,27^. Ward et al. (2020)^25^ find that summer blocking occurred on 205 days (15%) during the 2003–2018 period using used from Modern-Era Retrospective Analysis for Research and Applications, version 2 (MERRA-2) reanalysis data. The net radiative effects of summer blocking in the ablation zone, however, have yet to be directly observed.

**Fig. 3:**
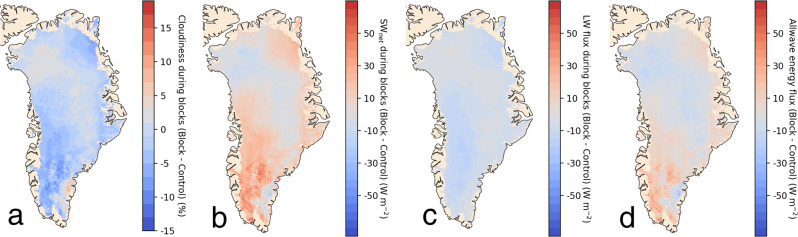
Radiative effects of all summer atmospheric blocking events on the surface of the Greenland Ice Sheet relative to mean (2003–2020) conditions. (**a**) cloudiness; (**b**) net shortwave radiative fluxes; (**c**) downward longwave radiative fluxes; (**d**) allwave radiative fluxes. Blocking days, which were provided by Ward et al.^25^, have a net positive (warming) effect on radiation receipt at the surface of the ablation zone.

We investigate the radiative effects of atmospheric blocking events by combining our high-resolution CRE product with blocking days identified by Ward et al. (2020)^25^ who locate blocking events into four quadrants (NW, NE, SE, SW) based on local maximum 500 hPa geopotential height anomalies. Depending on where the blocking is centered, we find that blocking events reduce cloudiness in the ablation zone by 0.2–6.4% percentage points (Table S1). When blocking events are centered over Northwest, Northeast, and Southwest Greenland, net radiative fluxes in the ablation zone increase by +11.5 ± 3.1 W m^−2^, +4.9 ± 1.3 W m^−2^ and +4.0 ± 1.1 W m^−2^, respectively (Table S1; Fig. 3), relative to mean summer conditions. These increased radiative fluxes can be attributed to a relatively large increase in downward shortwave radiation combined with a smaller decrease in downward longwave radiation due to warmer near-surface air temperatures^25^ (Fig. 3). When blocking events are centered over Southeast Greenland, net radiative fluxes are actually reduced by −1.6 ± 0.4 W m^−2^, because the loss of shortwave shading over the narrow Southeast Greenland ablation zone is countered by enhanced cloudiness over the larger Southwest and Northwest Greenland ablation zones (Fig. S5). Generally, these findings signify that the radiative effects of blocking events enhance meltwater production in the ablation zone, with their net effect varying depending on location.

### Cloud radiative effects in a warmer climate

**Fig. 4:**
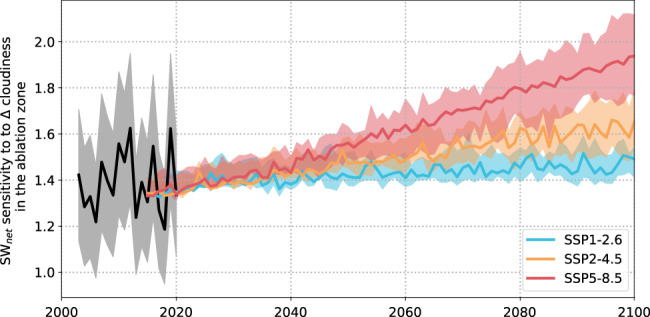
Sensitivity of net shortwave radiation to changes in summer cloudiness for a range of climate scenarios. Black line represents the current observed sensitivity derived from our high-resolution radiative flux products with shaded areas representing uncertainty (see Methods). Red, orange, and yellow lines represent projected changes in sensitivity for SSP1-2.6, SSP2-4.5, SSP5-8.5 scenarios based on the ensemble mean summer air temperature of 26 models from the CMIP6 experiment. Shaded areas represent the 25th and 75th percentile projections of all models.

In a warmer climate, the shortwave shading effect of clouds will strengthen in the ablation zone due to decreased surface albedo. To forecast future changes in ice sheet albedo, we used observed air temperature-albedo relationships to develop an empirical model that predicts albedo from future air temperature forecasts (see the section “Cloud radiative effects in the future”). By applying our model to a range of projected future air temperatures from CMIP6 global climate model simulations, we find that summer surface albedo in the ablation zone will darken by ~0.02–0.14 depending on the emissions scenario (Fig. S6). Interannual variations in cloudiness will therefore have a much greater effect on radiative fluxes on the ablation zone surface than they do presently. Under a high emissions scenario (SSP5-8.5), for example, we project that by 2100, a 1% change in cloudiness will alter net shortwave radiation by ±1.94 W m^−2^ (Fig. 4), which is much greater than its effect on downward longwave radiation (±1.55 W m^−2^; Fig. 2d). The difference between cloud shortwave shading and longwave trapping effects on the ablation zone (i.e. ±0.39 W m^−2^ in 2100 [1.55–1.94] versus ±0.16 W m^−2^ during our 2003–2020 study period [from Fig. 2; 1.39–1.55]) suggests that the sensitivity of ice sheet meltwater production to cloudiness will more than double in a future, warmer climate.

This increasing sensitivity of Greenland ablation zone energy balance to cloudiness implies that, in a warmer climate, atmospheric blocking events will enhance ice sheet melt even more than they do currently. By 2100, in an SSP5-8.5 scenario, we compute that the same blocking events centered over Northwest, Northeast, and Southwest Greenland will increase net radiation fluxes in the ablation zone by +15.7 ± 4.2, +6.6 ± 1.8, and +5.3 ± 1.4 W m^−2^ respectively, relative to mean 2100 conditions (Table S1). An overall ~34% increased radiative impact of atmospheric blocking events compared with the current study period (+4.7 for 2003–2020 period vs. +6.3 W m^−2^ by 2100 in SSP5-8.5), together with enhanced sensible heat fluxes from warm air advection^25^, will make these anomalous circulation events extremely potent for ice sheet melt in the future. It is therefore critical that global climate models used to forecast future ice mass loss accurately represent the frequency and duration of atmospheric blocking events over the Greenland Ice Sheet.

To conclude, we used machine learning to merge accurate Cloudsat/CALIPSO cloud profiling retrievals with high temporal and spatial resolution MODIS visible-infrared imagery, enabling retrieval and investigation of fine-scale CRE variations across the Greenland Ice Sheet surface from 2003 to 2020. The high spatial resolution (1 × 1 km) of our CRE product represents a major advance for understanding interactions between clouds and specific areas of the ice sheet surface. Previous observational studies of CRE were limited to either very coarsely gridded satellite products (e.g. 2 × 2°) or sparsely located in situ instruments, most of which are located in the accumulation zone. Our high-resolution spatially-continuous product overcomes these limitations, thus enabling systematic assessment of radiative fluxes over the entire Greenland Ice Sheet, including the ablation zone where the vast majority of ice sheet meltwater runoff is produced.

Analysis of our fine-scale CRE product reveals an important emerging role for clouds on future meltwater production on the Greenland Ice Sheet surface. During our 2003−2020 study period, the competing radiative impacts of clouds (i.e. longwave warming vs. shortwave cooling) approximately compensated over the ablation zone, signifying that clouds currently have a small regulatory role on meltwater runoff production. But as the ablation zone darkens^2^, the shortwave shading effect of clouds will become increasingly critical for determining meltwater production and associated ice sheet contributions to global sea-level rise. By the century’s end, we project that surface energy balance in the Greenland Ice Sheet ablation zone will be twice as sensitive to clouds than it is today and that atmospheric circulation patterns that suppress clouds (i.e. blocking events) will enhance meltwater production ~34% more than they already do.

## Methods

### General approach

Our general approach for investigating the impacts of clouds on Greenland Ice Sheet mass balance from satellite remote sensing observations and machine learning is outlined here with further details provided in the section “Prediction of cloud enhancement factors”. Central to our approach is the computation of CRE which describes the instantaneous effect of clouds on the surface energy budget relative to clear skies^28^:

1 $${{{{{{\rm{CRE}}}}}}}_{{{{{{\rm{SW}}}}}}}=({{{{{{\rm{SW}}}}}}}_{\downarrow {{{{{\rm{all}}}}}}-{{{{{\rm{sky}}}}}}}-{{{{{{\rm{SW}}}}}}}_{\downarrow {{{{{\rm{clear}}}}}}-{{{{{\rm{sky}}}}}}}).\,(1-\alpha )$$

2 $${{{{{{\rm{CRE}}}}}}}_{{{{{{\rm{LW}}}}}}}=({{{{{{\rm{LW}}}}}}}_{\downarrow {{{{{\rm{all}}}}}}-{{{{{\rm{sky}}}}}}}-{{{{{{\rm{LW}}}}}}}_{\downarrow {{{{{\rm{clear}}}}}}-{{{{{\rm{sky}}}}}}})$$

3 $${{{{{{\rm{CRE}}}}}}}_{{{{{{\rm{NET}}}}}}}={{{{{{\rm{CRE}}}}}}}_{{{{{{\rm{SW}}}}}}}+{{{{{{\rm{CRE}}}}}}}_{{{{{{\rm{LW}}}}}}}$$

where SW_↓all−sky_ and LW_↓all−sky_ are the downward shortwave and longwave fluxes at the surface during all-sky conditions, SW_↓clear−sky_ and LW_↓clear−sky_ are the downward shortwave and longwave fluxes that would occur at the surface in the absence of clouds, and α is the surface albedo. A positive CRE_NET_ (in W m^−2^) indicates net cloud warming at the surface, whereas a negative CRE_NET_ indicates net cloud cooling.

We first derive CRE_NET_ using high-quality all-sky and clear-sky radiative flux observations from CloudSat/CALIPSO and surface albedo observations from MODIS. Yet, while this approach may be accurate, CloudSat/CALIPSO has a relatively narrow ground footprint (~1.4 km), long repeat interval (16 days), and short timespan (2006−2010) which limits its usefulness of for investigating CRE at fine spatiotemporal resolution over long time periods^16,18^. To overcome this, we train a machine learning model, using CloudSat/CALIPSO observations, to predict CRE from passive visible-infrared satellite observations (i.e. from MODIS). This approach involves computation of a cloud enhancement factor (F) from CloudSat/CALIPSO given as:

4 $${{{\rm{F}}}}_{{{\rm{SW}}}} = {\frac {{{{\rm{SW}}}}_{\downarrow {{{\rm{all}}}}-{{{\rm{sky}}}}}} {{{{\rm{SW}}}}_{\downarrow {{{\rm{clear}}}}-{{{\rm{sky}}}}}}}$$

and

5 $${{{\rm{F}}}}_{{{\rm{LW}}}} = {\frac {{{{\rm{LW}}}}_{\downarrow {{{\rm{all}}}}-{{{\rm{sky}}}}}} {{{{\rm{LW}}}}_{\downarrow {{{\rm{clear}}}}-{{{\rm{sky}}}}}}}$$

The cloud enhancement factor represents the change in downward shortwave or longwave radiation due to clouds relative to clear skies^7^. This value depends upon cloud optical thickness, phase, temperature, and height, as well as solar zenith angle for shortwave fluxes and near-surface air temperature for longwave fluxes^7,29^. Our supervised machine learning algorithm is trained to predict F_SW_ and F_LW_ from cloud properties derived from passive visible-infrared sensors such as MODIS. Since MODIS has much wider spatial coverage and shorter repeat intervals (i.e. sub-daily) than CloudSat/CALIPSO, this approach enables computation of observational based CRE at finer spatial resolution and longer time-scales (2003−2020 vs. 2006–2010) than was previously possible. Further details on our approach are provided in the section “Prediction of cloud enhancement factors”.

### Data

We derived high-quality downward all-sky and clear-sky radiative fluxes at the surface from the CloudSat/CALIPSO Level-2 ‘Fluxes and Heating Rates’ (2B-FLXHR-LIDAR) product which was the first to use active remotely sensed cloud observations to retrieve surface radiative fluxes at a global scale^30,31^. This product incorporates (1) cloud and aerosol observations from CloudSat/CALIPSO; (2) ancillary temperature and humidity profiles from ECMWF atmospheric re-analyses; and (3) International Geosphere-Biosphere Programme surface albedo/emissivity data to constrain broadband radiative fluxes using the two-stream radiative transfer model “BugsRad”^31^. Since our study is focused on the summer, we only use 2B-FLXHR-LIDAR data acquired during June, July, and August. We use Release 05 version of the 2B-FLXHR-LIDAR product which provides a more robust partitioning of ice-only and liquid-bearing clouds than Release 04^7^. The radiative fluxes of the 2B-FLXHR-LIDAR algorithm have been extensively evaluated^7,31^ and previously used to study cloud impacts on surface energy budgets in the polar regions^7,11,18^.

We derived summer cloud properties from the MODIS Level-2 Cloud product “MYD06_L2” Collection 6.1. This product uses remotely sensed infrared, visible and near-infrared reflected radiances to derive cloud properties such as cloud top temperature, cloud top height, effective emissivity, cloud phase (ice vs. water), cloud optical thickness, and effective particle radius^19^. These parameters are available at a spatial resolution of either 1 km or 5 km resolution (at nadir). Since NASA’s Aqua satellite orbits within two to three minutes of CloudSat/CALIPSO in the A-Train constellation, cloud properties from the MYD06_L2 product are near-coincident with the 2B-FLXHR-LIDAR product. We also derived surface albedo from the MODIS daily snow cover product, MOD10A1 Collection 6.1.^2^. These data were averaged to provide mean summer albedo for the Greenland Ice Sheet at 1 × 1 km grid cell resolution. Since satellite measurements of albedo are acquired under clear-sky conditions and snow/ice albedo is known to be higher under cloud cover, we applied a correction to our clear-sky albedo climatologies following Key et al.^32^. To this, we multiplied the mean cloud effect (+0.05) with the cloud fraction of each grid cell (Fig. 1a) and added this value to the clear-sky albedo climatology. Finally, hourly near-surface (2 m) air temperatures were derived from ERA5 climate reanalysis^33^.

### Prediction of cloud enhancement factors

We predicted cloud enhancement factors for each MODIS pixel using a Random Forests regression algorithm trained from CloudSat/CALIPSO radiative flux observations. The approach was introduced in the section “General approach” but the specific steps are described here:

#### Response variable

We first computed cloud enhancement factors (F_SW_ and F_LW_) from all CloudSat/CALIPSO downward all-sky and clear-sky radiative flux observations acquired over the Greenland Ice Sheet between 13 June 2006 and 18 August 2010 (Eqs. 4 and 5).

#### Predictive variables

For each CloudSat/CALIPSO cloud enhancement factor (*n* = 317,614), we acquired coincident cloud properties from the MYD06_L2 product and near-surface temperatures from ERA5 climate reanalysis using a nearest neighbors approach (*k* = 1). Cloud properties used to predict F_SW_ include cloud optical thickness, cloud top pressure, cloud effective radius, cloud top temperature, cloud top height, and cloud water path from MODIS, and near-surface air temperature from ERA5. Cloud properties used to predict F_LW_ include cloud optical thickness, cloud top pressure, cloud effective radius, cloud top temperature, cloud top height, cloud water path, and cloud phase from MODIS and near-surface air temperature from ERA5. Since downward shortwave all-sky radiative fluxes are highly dependent on solar zenith angle, we grouped the training data into 5° bins (between 35 and 80°) before statistical modeling.

#### Statistical modeling

For each group of training data (ten groups for F_SW_ and one group for F_LW_), we fitted a Random Forests regression model with 100 trees to a random sample containing 50% of the training data (*n* = 158,807 for F_LW_ and *n* = ~15,000 for each F_SW_ group). We then used the regression models (which depend on solar zenith angle for the F_SW_ model) to predict F_SW_ and F_LW_ for every valid pixel in every MYD06_L2 product acquired over the Greenland Ice Sheet during the 2003–2020 period. The importance of each variable for predicting F_SW_ and F_LW_ is provided in Table S2 and S3. We evaluated our models using the other 50% of the data that was not used for training (see the section “Error budget”).

### Computation of metrics

For each predicted F_SW_ and F_LW_, we derived corresponding downward clear-sky radiative fluxes from ERA5 reanalysis. To account for possible bias in the ERA5 reanalysis, we first performed linear regression between 2B-FLXHR-LIDAR and ERA5 reanalysis clear-sky radiative fluxes and calibrated ERA5 clear-sky fluxes using the slope and intercept of this function (Fig. S7). We then combined clear-sky fluxes with the aforementioned cloud enhancement factors (F_SW_ and F_LW_) to compute downward all-sky fluxes (using Eqs. 4 and 5).

Next, we combined these downward all-sky fluxes with downward clear-sky fluxes and mean summer surface albedo derived in Ryan et al. (2019)^2^ to compute summer CRE_SW_, CRE_LW_ and CRE_NET_ (using Eqs. 1, 2 and 3) for every pixel in every MYD06_L2 product acquired over the Greenland Ice Sheet during the 2003–2020 period. We also derive a “cloudiness” metric which was defined as the percentage reduction in shortwave radiation at the surface due to clouds (Eq. 6).

6 $${{{{{\rm{Cloudiness}}}}}} = \frac {\left({{{{{{\rm{SW}}}}}}}_{\downarrow {{{{{\rm{clear}}}}}}-{{{{{\rm{sky}}}}}}}-{{{{{{\rm{SW}}}}}}}_{\downarrow {{{{{\rm{all}}}}}}-{{{{{\rm{sky}}}}}}}\right)}{{{{{{{\rm{SW}}}}}}}_{\downarrow {{{{{\rm{clear}}}}}}-{{{{{\rm{sky}}}}}}}}$$

### Radiative flux climatologies

Our satellite-derived CRE observations reflect cloud conditions at the time of satellite overpass which for parts of South Greenland is only once or twice per day (Fig. S8). This biases our radiative flux climatologies high because all-sky radiative fluxes are derived during daytime. To overcome this issue, we computed the mean difference between hourly and mean daily shortwave radiative fluxes in ERA5 between 2003 and 2020 for every ERA5 grid cell on the Greenland Ice Sheet. We then subtracted this correction from our instantaneous all-sky and clear-sky shortwave radiative fluxes. After calibration, our satellite-derived all-sky and clear-sky shortwave radiative fluxes represent daily means. Downward longwave radiation mostly depends on cloudiness and does not exhibit any predictable diurnal cycle in AWS data or ERA5 reanalysis, so we assumed our instantaneous satellite-derived all-sky and clear-sky longwave radiative fluxes represent daily means which introduces an uncertainty of ±11.6 W m^−2^ (Fig. S9).

We bilinearly resampled our products to a standard 1 × 1 km study grid used by the Greenland Surface Mass Balance Intercomparison Project (GrSMBMIP)^34^. We separated our results into the ablation and accumulation zones based on a bare ice presence index threshold described by Ryan et al. (2019)^2^. The ablation zone was defined as all areas of the ice sheet where the bare ice is present for more than 10% of the record-setting melt summer of 2012 with the rest of the ice sheet classified as the accumulation zone. The ablation zone represents 14.8% of the ice sheet area. We average our sub-daily CRE products for each summer (June, July, and August) during the 2003–2020 period, providing an 18-year record of cloud conditions and radiative effects over the Greenland Ice Sheet.

### Error budget

The uncertainties in our MODIS-derived all-sky radiative fluxes are a combination of uncertainties in predicted cloud enhancement factors (F_SW_ and F_LW_) and clear-sky radiative fluxes (Eqs. 4 and 5). We evaluated our predicted cloud enhancement factors against high-quality cloud enhancement factors derived from CloudSat/CALIPSO observations (*n* = 158,807 for F_LW_ and *n* = ~15,000 for F_SW_) that were not used to train the machine learning algorithm. We found a mean absolute error (MAE) of ±0.075 (±11.7%) for predicted F_SW_ (Fig. S10a) and ±0.036 (±2.6%) for predicted F_LW_ (Fig. S10b).

We evaluated our clear-sky radiative fluxes by comparing the clear-sky fluxes from ERA5 reanalysis with the 2B-FLXHR-LIDAR product. We find a MAE of ±20.8 W m^−2^ (±5.6%) between clear-sky shortwave fluxes (Fig. S7a) and a MAE ± 13.7 W m^−2^ (±6.7%) for the clear-sky longwave fluxes (Fig. S7b and S9). We assigned an uncertainty of ±0.03 (±5.8%) to our MODIS-derived summer albedo product based on previous comparisons with surface albedo data from PROMICE AWS^35^. When these sources of error are combined, we compute an uncertainty of ±17.3% for downward SW_all-sky_, ±9.3% for downward Lw_all-sky_, ±23.9% for CRE_SW_, ± 11.9% for CRE_LW_, and ±26.7% for CRE_NET_.

We carried out an independent check on our satellite-derived product by comparing it to high-quality CRE observations acquired by in situ instruments at Summit Station (72.68 °N, −38.58 °W)^13^. Between the period 2011–2013, Miller et al. (2015)^13^ observed a CRE_NET_ of +35.3 W m^−2^ during the summer which is very similar to what we find over the same time period (+32.3 ± 8.7 W m^−2^).

### Cloud radiative effects in the future

While we find no evidence that cloudiness in the ablation zone will substantially change as the climate warms, the albedo of the ablation zone will likely reduce in a warmer climate due to migrating snowlines, algal growth, and enhanced meltwater production. To forecast future albedo in the ablation zone, we developed an empirical model that predicts albedo for each grid cell based on a reference albedo grid (2015–2020) and near-surface air temperatures from ERA5 climate reanalysis and 26 models from the CMIP6 climate model experiment. We first linearly regressed mean summer albedo from MODIS with downscaled air temperature from ERA5 for each 1 × 1 km grid cell for the 2003–2020 period. ERA5 summer air temperatures were downscaled onto the target 1 km DEM by using the Local Inverse Distance Weighting (IDW) Lapse Algorithm from the MeteoIO library^36^. Regional lapse rates were calculated from all ERA5 grid points within 100 km distance from the target grid point. The obtained lapse rates were used to remove the elevation gradient from the ERA5 grid points. We then interpolated the residuals over the 1 km target DEM by inverse distance weighting using a scale of 1 km, and the power value alpha set to 1. Finally, the locally calculated lapse rate is used again to add the elevation gradient to the 1 km DEM to the interpolated grid.

After downscaling, non-significant (>0.05) and unrealistic relationships (i.e. positive correlations) were removed from analysis. We then computed the mean slope of these linear relationships within nine temperature bands (Fig. S11). This means that the sensitivity of grid cell albedo to air temperature is dynamic in response to a warming climate. For example, the albedo of a grid cell that is permanently snow-covered during the 2015–2020 reference period might not be initially very sensitive to warmer summer air temperatures, because the only increasing snow grain size can modify the grid cell albedo. However, if sufficient warming (and melting) occurs, bare ice could be exposed in the same grid cell, dramatically reducing the albedo. Our empirical albedo scheme would capture the addition of this process by placing the grid cell into the next, more sensitive, temperature band that represent grid cells whose albedos respond to both snow grain size evolution and bare ice exposure. Our empirical model therefore captures expected non-linearity between air temperature and albedo. We evaluated our model using temperature data from before our reference period (i.e. 2003–2014). We find a strong correlation between observed and predicted albedo (*R*^2^ = 0.83) with an accuracy of ±0.04 (Fig. S12).

We forecasted the albedo of the ice sheet to 2100 using near-surface air temperatures provided by the CMIP6 climate model experiment for three Shared Socioeconomic Pathways (SSP) (SSP1-2.6, SSP2-4.5, SSP3-7.0 and SSP5-8.5). We used 26 models, namely: ACCESS-CM2, ACCESS-ESM1-5, CESM2-WACCM, CESM2, CMCC-CM2-SR5, CNRM-CM6-1-HR, CNRM-CM6-1, CNRM-ESM2-1, CanESM5-CanOE, CanESM5, EC-Earth3-Veg, EC-Earth3, GFDL-ESM4, GISS-E2-1-G, INM-CM4-8, INM-CM5-0, IPSL-CM6A-LR, KACE-1-0-G, MIROC-ES2L, MIROC6, MPI-ESM1−2-HR, MPI-ESM1-2-LR, MRI-ESM2-0, NorESM2-LM, NorESM2-MM, and UKESM1-0-LL. This ensemble has a size and breadth of models that is comparable to the ensembles used in parts of the IPCC AR6 WG1 report. For each model we resampled near-surface air temperatures onto a common study grid using bilinear interpolation. For each model we also normalized air temperature by subtracting the mean summer ice sheet air temperature to account for any temperature bias. We then derived albedo for each grid cell based on the temperature deviation from a 2015–2020 reference period and the empirical albedo scheme described above. During this process, grid cell albedo is prevented from going below 0.3, below which would be an unrealistically low albedo for bare ice (Fig. S6). Finally, we computed the linear relationship between cloudiness (see the section “ Computation of metrics”) and SW_net_ [i.e. SW_↓all−sky_ * (1 – α)] based on the forecasted albedo in the ablation zone. This enabled us to assess the sensitivity of ice sheet meltwater production to changing cloudiness in a future, warmer climate (Fig. 4).

## Supplementary information

Supplementary Information

## Data Availability

The cloud radiative effect climatologies produced in this study are at: https://zenodo.org/record/6582251#.YpkhzBPMLX0. The raw 2B-FLXHR-LIDAR data can be accessed from the CloudSat Data Processing Center at: http://www.cloudsat.cira.colostate.edu/data-products/level−2b/2b-flxhr-lidar. The raw MYD06 L2 data can be accessed from NASA EarthData at: https://earthdata.nasa.gov/. ERA5 reanalysis data can be accessed from the Copernicus Climate Data Store at: https://cds.climate.copernicus.eu/cdsapp#!/dataset/reanalysis-era5-pressure-levels?tab=overview. Data used to downscale the ERA5 climate data are available at: https://zenodo.org/record/6456281#.YlbwpNPMLX0.

## Code availability

All code required to reproduce the results of this study are available at: https://zenodo.org/record/6608977#.Ypkj1BPMLX0. Additional code used for downscaling ERA5 climate data are available at: https://zenodo.org/record/6456281#.YpkjphPMLX1.

## References

[1] Chen X (2017). The increasing rate of global mean sea-level rise during 1993–2014. Nat. Clim. Change.

[2] Ryan, J. C. et al. Greenland Ice Sheet surface melt amplified by snowline migration and bare ice exposure. *Sci. Adv*. **5**, eaav3738 (2019). 10.1126/sciadv.aav3738 PMC6402853 30854432

[3] Steger CR, Reijmer CH, Van Den Broeke MR (2017). The modelled liquid water balance of the Greenland Ice Sheet. Cryosphere.

[4] Hofer S, Tedstone AJ, Fettweis X, Bamber JL (2019). Cloud microphysics and circulation anomalies control differences in future Greenland melt. Nat. Clim. Change.

[5] Hofer, S., Tedstone, A. J., Fettweis, X. & Bamber, J. L. Decreasing cloud cover drives the recent mass loss on the Greenland Ice Sheet. *Sci. Adv*. **3**, e1700584 (2017). 10.1126/sciadv.1700584 PMC5489271 28782014

[6] Izeboud, M. et al. The Spatiotemporal Variability of Cloud Radiative Effects on the Greenland Ice Sheet Surface Mass Balance. *Geophys. Res. Lett*. **47**, e2020GL087315 (2020).

[7] Van Tricht K (2016). Clouds enhance Greenland ice sheet meltwater runoff. Nat. Commun..

[8] Lenaerts JTM, Van Tricht K, Lhermitte S, L’Ecuyer TS (2017). Polar clouds and radiation in satellite observations, reanalyses, and climate models. Geophys. Res. Lett..

[9] Bennartz R (2013). July 2012 Greenland melt extent enhanced by low-level liquid clouds. Nature.

[10] Miller NB (2018). Process-Based Model Evaluation Using Surface Energy Budget Observations in Central Greenland. J. Geophys. Res. Atmospheres.

[11] Lenaerts, J. T. M., Gettelman, A., Van Tricht, K., van Kampenhout, L. & Miller, N. B. Impact of Cloud Physics on the Greenland Ice Sheet Near-Surface Climate: A Study With the Community Atmosphere Model. *J. Geophys. Res. Atmospheres* **125**, 1–25 (2020).

[12] Cullather RI, Nowicki SMJ (2018). Greenland Ice Sheet surface melt and its relation to daily atmospheric conditions. J. Clim..

[13] Miller NB (2015). Cloud radiative forcing at Summit, Greenland. J. Clim..

[14] Shupe MD (2013). High and dry: New observations of tropospheric and cloud properties above the greenland ice sheet. Bull. Am. Meteorol. Soc..

[15] Wang W, Zender CS, van As D (2018). Temporal Characteristics of Cloud Radiative Effects on the Greenland Ice Sheet: Discoveries From Multiyear Automatic Weather Station Measurements. J. Geophys. Res. Atmospheres.

[16] Wang W, Zender CS, van As D, Miller NB (2019). Spatial Distribution of Melt Season Cloud Radiative Effects Over Greenland: Evaluating Satellite Observations, Reanalyses, and Model Simulations Against In Situ Measurements. J. Geophys. Res. Atmospheres.

[17] Kay JE, L’Ecuyer T, Gettelman A, Stephens G, O’Dell C (2008). The contribution of cloud and radiation anomalies to the 2007 Arctic sea ice extent minimum. Geophys. Res. Lett..

[18] Kay JE, L’Ecuyer T (2013). Observational constraints on Arctic Ocean clouds and radiative fluxes during the early 21st century. J. Geophys. Res. Atmospheres.

[19] Platnick S (2003). The MODIS cloud products: Algorithms and examples from Terra. IEEE Trans. Geosci. Remote Sens..

[20] Reichstein M (2019). Deep learning and process understanding for data-driven Earth system science. Nature.

[21] Gislason PO, Benediktsson JA, Sveinsson JR (2006). Random forests for land cover classification. Pattern Recognit. Lett..

[22] Jung M (2010). Recent decline in the global land evapotranspiration trend due to limited moisture supply. Nature.

[23] Landschützer P (2013). A neural network-based estimate of the seasonal to inter-annual variability of the Atlantic Ocean carbon sink. Biogeosciences.

[24] Ryan, J. C. et al. Dark zone of the Greenland Ice Sheet controlled by distributed biologically-active impurities. *Nat. Commun*. **9**, 1065 (2018). 10.1038/s41467-018-03353-2 PMC5852041 29540720

[25] Ward, J. L., Flanner, M. G. & Dunn-Sigouin, E. Impacts of Greenland Block Location on Clouds and Surface Energy Fluxes Over the Greenland Ice Sheet. *J. Geophys. Res. Atmospheres* **125**, e2020JD033172 (2020).

[26] McLeod JT, Mote TL (2016). Linking interannual variability in extreme Greenland blocking episodes to the recent increase in summer melting across the Greenland ice sheet. Int. J. Climatol..

[27] Ballinger TJ (2018). Greenland coastal air temperatures linked to Baffin Bay and Greenland Sea ice conditions during autumn through regional blocking patterns. Clim. Dyn..

[28] Ramanathan V (1989). Cloud-Radiative Forcing and Climate: Results from the Earth Radiation Budget Experiment. Science.

[29] Shupe MD, Intrieri JM (2004). Cloud Radiative Forcing of the Arctic Surface: The Influence of Cloud Properties, Surface Albedo, and Solar Zenith Angle. J. Clim..

[30] L’Ecuyer TS, Wood NB, Haladay T, Stephens GL, Stackhouse PW (2009). Impact of clouds on atmospheric heating based on the R04 CloudSat fluxes and heating rates data set. J. Geophys. Res. Atmospheres.

[31] Henderson DS, L’Ecuyer T, Stephens G, Partain P, Sekiguchi M (2013). A Multisensor Perspective on the Radiative Impacts of Clouds and Aerosols. J. Appl. Meteorol. Climatol..

[32] Key JR, Wang X, Stoeve JC, Fowler C (2001). Estimating the cloudy-sky albedo of sea ice and snow from space. J. Geophys. Res. Atmospheres.

[33] Hersbach, H. et al. The ERA5 global reanalysis. *Q. J. R. Meteorol. Soc*. **146**, 1999–2049 (2020).

[34] Fettweis, X. et al. GrSMBMIP: intercomparison of the modelled 1980–2012 surface mass balance over the Greenland Ice Sheet. *Cryosphere* **14**, 3935–3958 (2020).

[35] Ryan, J. C. et al. How robust are in situ observations for validating satellite-derived albedo over the dark zone of the Greenland Ice Sheet? *Geophys. Res. Lett*. **44**, 6218–6225 (2017).

[36] Bavay M, Egger T (2014). MeteoIO 2.4.2: a preprocessing library for meteorological data. Geosci. Model Dev..

