# Supplementary material for: Decreasing surface albedo signifies a growing importance of clouds for Greenland Ice Sheet meltwater production
Source: Nat Commun. 2022 Jul 21;13:4205. doi: 10.1038/s41467-022-31434-w (PMC9304359; doi:10.1038/s41467-022-31434-w)
Supplement: Supplementary file 1 — Supplementary Information [file 41467_2022_31434_MOESM1_ESM.pdf]

Decreasing surface albedo signifies a growing importance of clouds for Greenland Ice Sheet meltwater production

J. C. Ryan[1], L. C. Smith[2,3], S. W. Cooley[1], B. Pearson[4], N. Wever[5], E. Keenan[5], and J. Lenaerts[5]

[1]Department of Geography, University of Oregon, USA
[2]Institute at Brown for Environment and Society, Brown University, USA
[3]Department of Earth, Environmental and Planetary Sciences, Brown University, USA
[4]College of Earth, Ocean, and Atmospheric Sciences, Oregon State University, USA
[5]Department of Atmospheric and Oceanic Sciences, University of Colorado, Boulder, CO, USA

Extended figures and tables

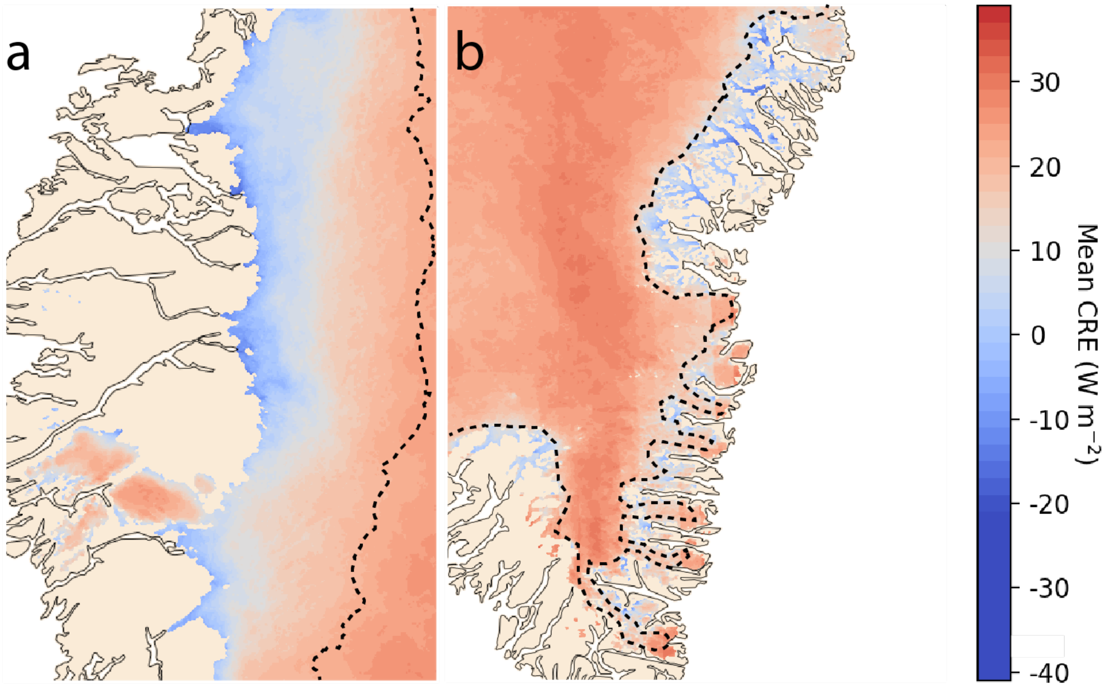

Figure S1: Maps showing mean summer cloud radiative effect (CRE) for the 2003-2020 study period. (a) Southwest Greenland, (b) Southeast and South Greenland. Dashed line represents boundary between the ablation and accumulation zones. The ablation zone was defined as all areas of the ice sheet where the bare ice is present for more than 10% of the record-setting melt summer of 2012 with the rest of the ice sheet classified as the accumulation zone.

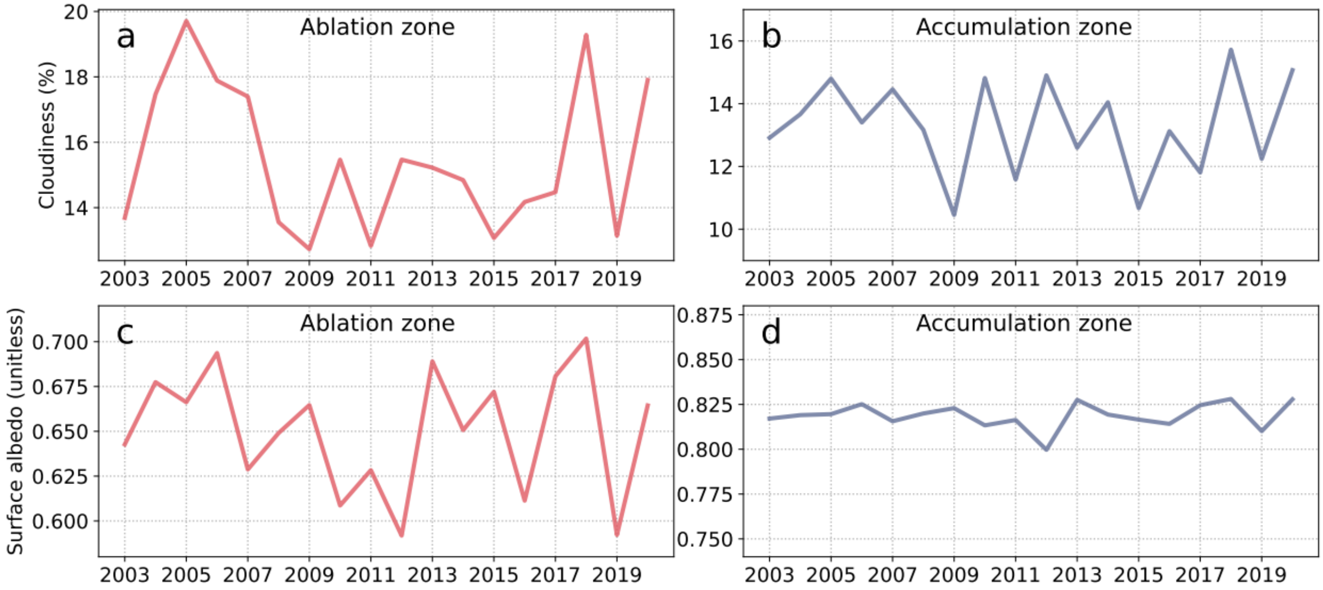

Figure S2: Interannual variations in summer cloudiness and surface albedo. (a) Cloudiness in the ablation zone, (b) cloudiness in the accumulation zone, (c) surface albedo in the ablation zone, (d) surface albedo in the accumulation zone. Note that the y-axes are scaled so the ranges are the same.

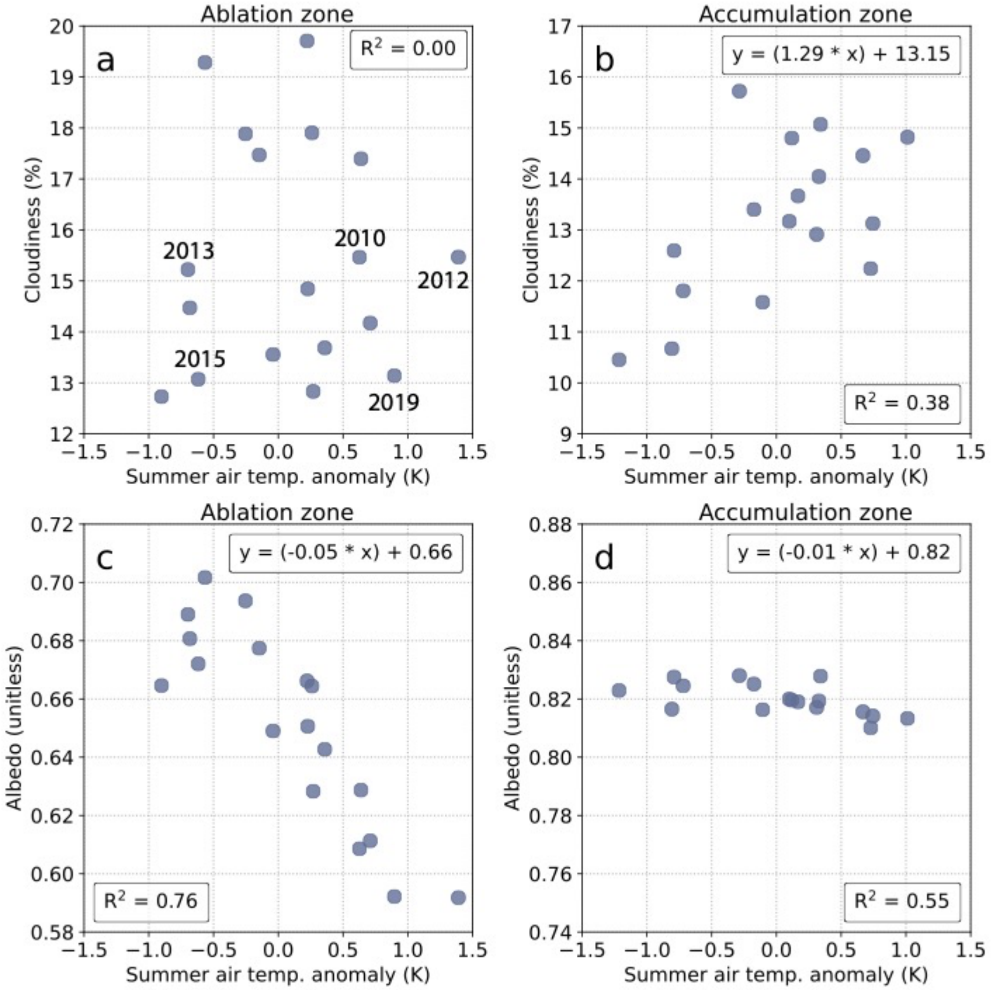

Figure S3: Relationships between mean summer near-surface air temperature, cloudiness, and surface albedo. (a) Sumer air temperatures exhibit no significant correlation with cloudiness in the ablation zone. Some intense (2007, 2010, 2012) and less intense (2013, 2015) melt-years are labeled to demonstrate that warmer summers are no cloudier/clearer than cooler summers. (b) Summer air temperatures are strongly correlated with cloudiness in the accumulation zone. (c) Summer air temperatures are strongly correlated with surface albedo in the ablation zone. (d) same as (d) but for the accumulation zone. Note that the y-axes are scaled so the ranges are the same.

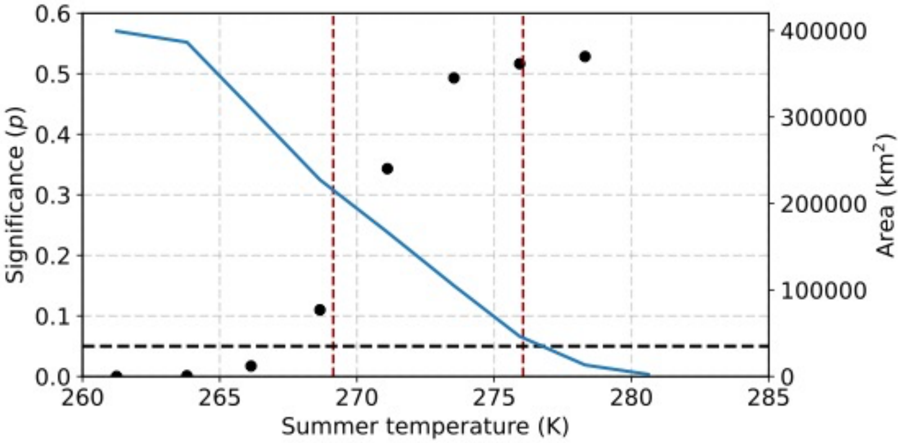

Figure S4: Plot showing how the significance (*p* value) of the linear relationship between mean summer air temperature and cloudiness changes with air temperature. Black dots represent significance, blue line represents ice sheet area, dashed black line represents the 0.05 significance. Dashed vertical red lines represent the 10th and 90th percentiles of ablation zone mean summer temperature. In cold regions of the ice sheet (i.e. accumulation zone) there is a significant positive correlation between air temperature and cloudiness, presumably due to phase transitions from ice to liquid water. In warmer regions (i.e. the ablation zone) this relationship becomes statistically insignificant, perhaps because most cloud water content has already transitioned from ice to liquid. This decoupling indicates that climate warming will *not* directly affect cloudiness in the ablation zone. We note that other factors such as changing circulation patterns could impact cloudiness in the ablation zone but assessment of these factors was considered beyond the scope of this study due to their current unpredictability.

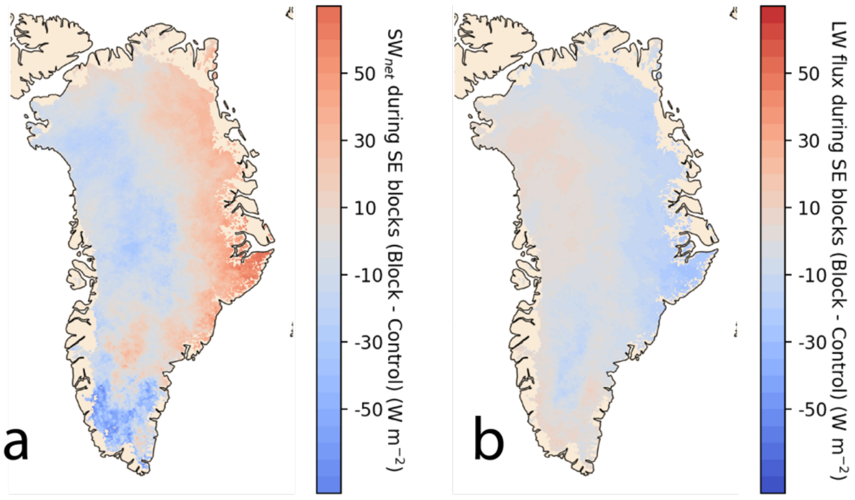

Figure S5: Radiative effects of summer atmospheric blocking events centered over SE Greenland on the surface of the Greenland Ice Sheet relative to mean (2003-2020) conditions: (a) net shortwave radiative fluxes; (b) downward longwave radiative fluxes. Net radiative fluxes are actually reduced by blocking events in SE Greenland because the loss of shortwave shading over the narrow Southeast Greenland ablation zone is countered by enhanced cloudiness over the larger Southwest and Northwest Greenland ablation zones.

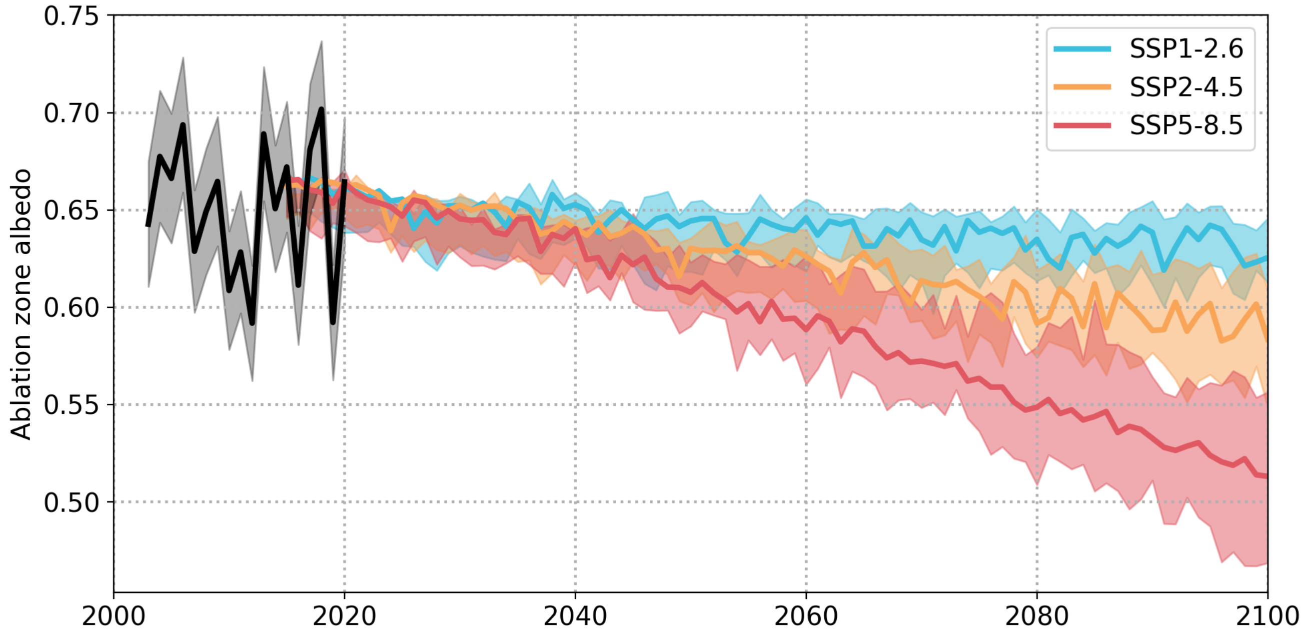

Figure S6: Future changes in albedo in the ablation zone in response to a range of climate scenarios. Albedo was computed using an empirical model that predicts albedo for each grid cell based on a reference albedo grid (2015-2020) and near-surface air temperatures from ERA5 climate reanalysis and 26 models from the CMIP6 climate model experiment (see Methods). Black line represents observed albedo derived from MODIS with shaded areas representing uncertainty. Red, orange, and yellow lines represent projected changes in albedo for SSP1-2.6, SSP2-4.5, SSP5-8.5 scenarios based on the ensemble mean summer air temperature of 26 models from the CMIP6 experiment. Shaded areas represent the interquartile range in projections of all models.

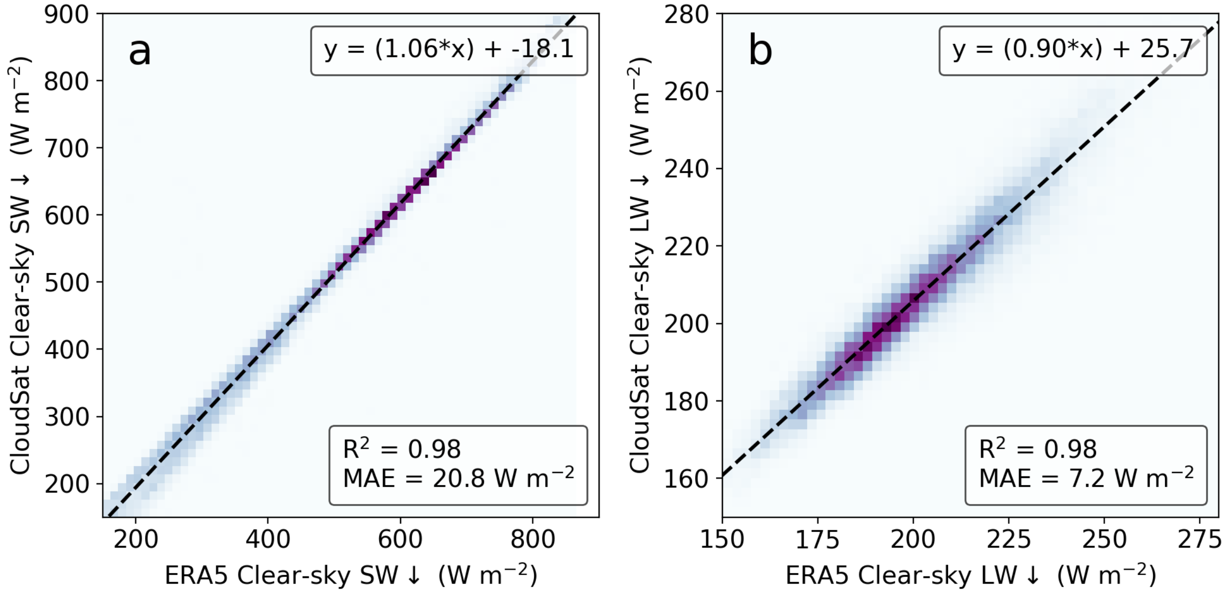

Figure S7: Comparison of CloudSat/CALIPSO and ERA5 clear-sky radiative fluxes. (a) Downward shortwave radiation and (b) downward longwave radiation.

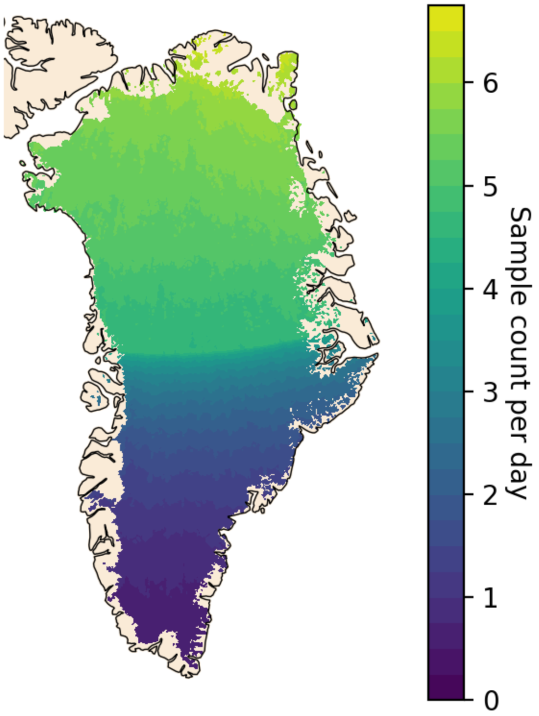

Figure S8: Map showing average number of MODIS retrievals across the ice sheet per day. Some areas of the ice sheet in North Greenland receive up to six MODIS observations per day while South Greenland only receives one or two.

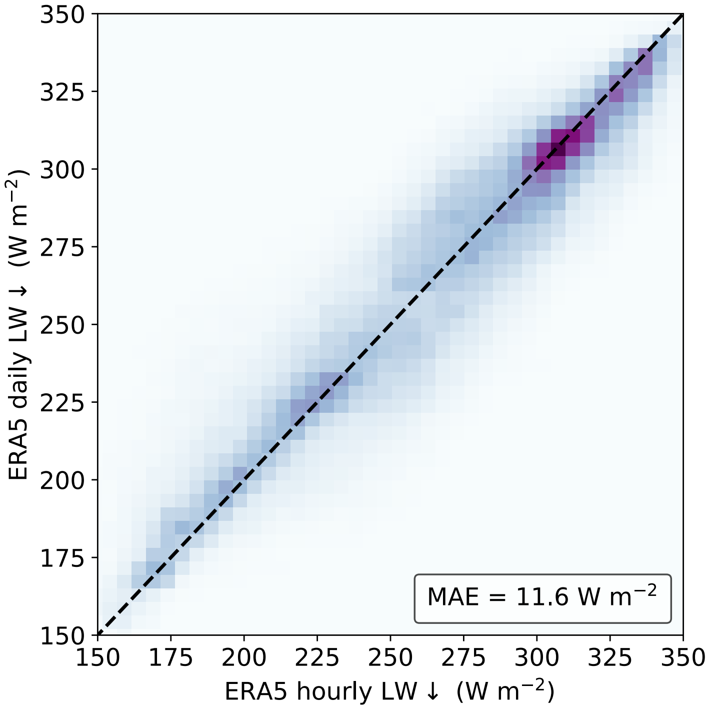

Figure S9: Relationship between daily and hourly downward LW radiation from ERA5. To produce this plot, we compared hourly values (n=24) to the corresponding daily mean. We carried out this analysis for 200 randomly distributed grid cells on the Greenland Ice Sheet during the summer for the 2003-2020 period.

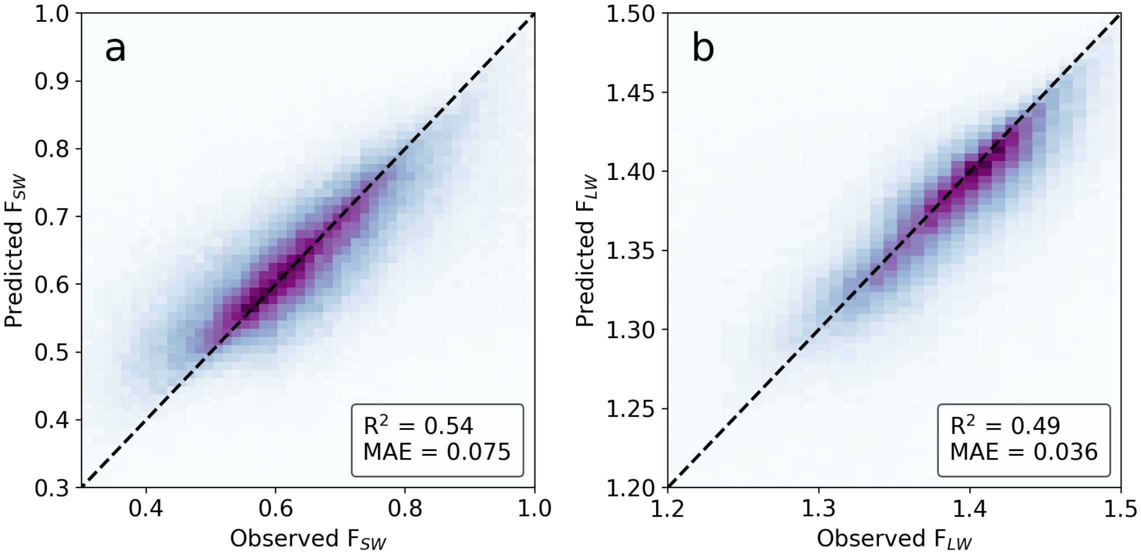

Figure S10: Evaluation of cloud enhancement factor (F) uncertainties. MAE represents mean absolute error. (a) Relationship between observed and predicted F_{SW}. (b) same as (a) but for F_{LW}.

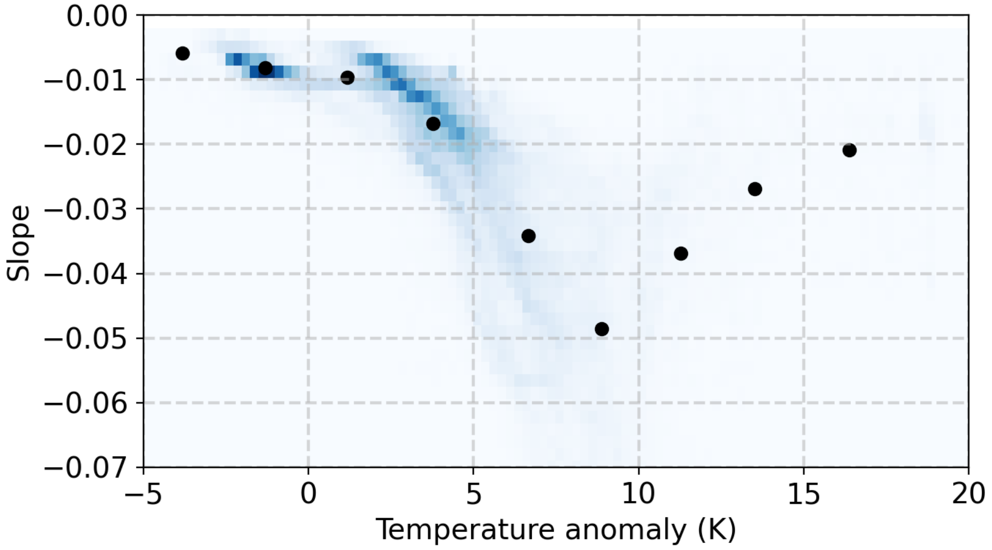

Figure S11: Slope (or sensitivity) between mean summer albedo and air temperature for each grid cell of the ice sheet for the 2003-2020 period. The y-axis (*temperature anomaly*) represents the difference between grid cell summer air temperature and ice sheet mean summer air temperature. Black dots represent the mean sensitivity within nine temperature bands (2.5 K range). In cold regions of the ice sheet, summer albedo is not particularly sensitive to summer air temperature, likely because the only way the air temperature can modify albedo is increasing snow grain size. In moderately warm regions of the ice sheet, the sensitivity between albedo and air temperatures increases. During the summer, this region is a mixture of bare ice and snow. Processes such as snowline migration and meltwater production therefore have a large impact on albedo. Finally, in very warm regions of the ice sheet, the sensitivity decreases again, likely because this region is bare ice for most of the summer. The only way that air temperature could affect albedo in this region is through enhanced algal growth and meltwater production, which do not affect albedo as much as snowline fluctuations.

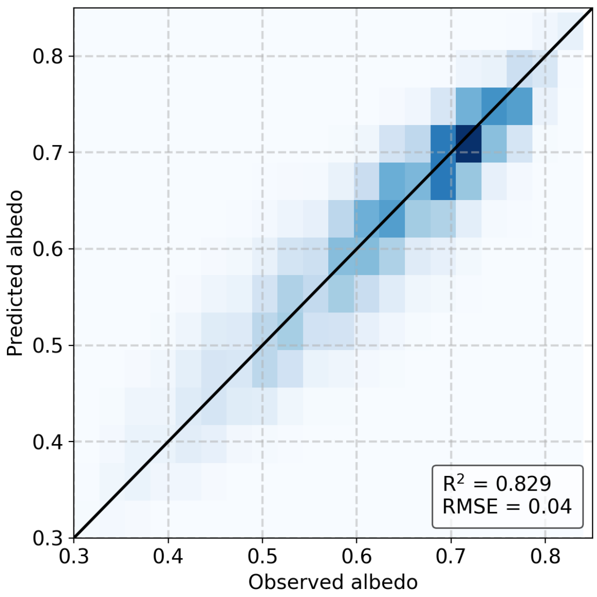

Figure S12: Relationship between observed albedo and that predicted by our empirical model for summer air temperatures during the 2003-2014 period.

**Ablation zone**	Block location			
	Southwest	Northwest	Northeast	Southeast
Cloudiness response (%)	-2.8	-5.7	-6.4	-0.2
Radiative effect (W m^{-2}) current (2003-2020)	+4.0	+11.5	+4.9	-1.6
Radiative effect (W m^{-2}) by 2100 (SSP1-2.6)	+4.2	+12.3	+5.3	-1.8
Radiative effect (W m^{-2}) by 2100 (SSP2-4.5)	+4.6	+13.5	+5.8	-2.0
Radiative effect (W m^{-2}) by 2100 (SSP5-8.5)	+5.3	+15.7	+6.6	-2.5

Table S1: Response of cloudiness and allwave radiative fluxes received at the surface of the Greenland Ice Sheet ablation zone in response to blocking events currently and by 2100 for three difference Shared Socioeconomic Pathways.

**Variable**	**Importance**
Near-surface air temperature	0.32
Cloud top temperature	0.18
Cloud optical thickness	0.14
Cloud effective radius	0.14
Cloud top height	0.10
Cloud water path	0.10
Cloud top pressure	0.03

Table S2: Importance of features for predicting SW cloud enhancement factors.

**Variable**	**Importance**
Near-surface air temperature	0.40
Cloud top temperature	0.14
Cloud effective radius	0.12
Cloud optical thickness	0.11
Cloud top height	0.09
Cloud water path	0.07
Cloud phase	0.04
Cloud top pressure	0.03

Table S3: Importance of features for predicting LW cloud enhancement factors.
